# Combinatory analysis of immune cell subsets and tumor-specific genetic variants predict clinical response to PD-1 blockade in patients with non-small cell lung cancer

**DOI:** 10.3389/fonc.2022.1073457

**Published:** 2023-02-09

**Authors:** Nikita Dutta, Anna Rohlin, Ella A. Eklund, Maria K. Magnusson, Frida Nilsson, Levent M. Akyürek, Per Torstensson, Volkan I. Sayin, Anna Lundgren, Andreas Hallqvist, Sukanya Raghavan

**Affiliations:** ^1^ Department of Microbiology and Immunology, Institute of Biomedicine, Sahlgrenska Academy, University of Gothenburg, Gothenburg, Sweden; ^2^ Department of Clinical Genetics and Genomics, Sahlgrenska University Hospital, Gothenburg, Sweden; ^3^ Department of Laboratory Medicine, Institute of Biomedicine, Sahlgrenska Academy, University of Gothenburg, Gothenburg, Sweden; ^4^ Department of Surgery, Institute of Clinical Sciences, Sahlgrenska Center for Cancer Research, University of Gothenburg, Gothenburg, Sweden; ^5^ Wallenberg Center for Molecular and Translational Medicine, University of Gothenburg, Gothenburg, Sweden; ^6^ Department of Oncology, Sahlgrenska University Hospital, Gothenburg, Sweden; ^7^ Department of Clinical Pathology, Institute of Biomedicine, Sahlgrenska University Hospital, Gothenburg, Sweden; ^8^ Department of Pulmonary Medicine, Skaraborg hospital, Skövde, Sweden; ^9^ Department of Clinical Immunology and Transfusion Medicine, Sahlgrenska University Hospital, Gothenburg, Sweden; ^10^ Department of Oncology, Institute of Clinical Sciences, Sahlgrenska Academy, University of Gothenburg, Gothenburg, Sweden

**Keywords:** non-small cell lung cancer, PD-1, TP53, KRAS, effector memory T cells

## Abstract

**Objectives:**

Immunotherapy by blocking programmed death protein-1 (PD-1) or programmed death protein-ligand1 (PD-L1) with antibodies (PD-1 blockade) has revolutionized treatment options for patients with non-small cell lung cancer (NSCLC). However, the benefit of immunotherapy is limited to a subset of patients. This study aimed to investigate the value of combining immune and genetic variables analyzed within 3–4 weeks after the start of PD-1 blockade therapy to predict long-term clinical response.

**Materials and methodology:**

Blood collected from patients with NSCLC were analyzed for changes in the frequency and concentration of immune cells using a clinical flow cytometry assay. Next-generation sequencing (NGS) was performed on DNA extracted from archival tumor biopsies of the same patients. Patients were categorized as clinical responders or non-responders based on the 9 months’ assessment after the start of therapy.

**Results:**

We report a significant increase in the post-treatment frequency of activated effector memory CD4^+^ and CD8^+^ T-cells compared with pre-treatment levels in the blood. Baseline frequencies of B cells but not NK cells, T cells, or regulatory T cells were associated with the clinical response to PD-1 blockade. NGS of tumor tissues identified pathogenic or likely pathogenic mutations in tumor protein P53, Kirsten rat sarcoma virus, Kelch-like ECH-associated protein 1, neurogenic locus notch homolog protein 1, and serine/threonine kinase 11, primarily in the responder group. Finally, multivariate analysis of combined immune and genetic factors but neither alone, could discriminate between responders and non-responders.

**Conclusion:**

Combined analyses of select immune cell subsets and genetic mutations could predict early clinical responses to immunotherapy in patients with NSCLC and after validation, can guide clinical precision medicine efforts.

## 1 Introduction

Anti-Programmed cell death protein-1 (PD-1) and anti-programmed death protein ligand 1 (PD-L1) treatment has received approval from the US Food and Drug Administration (FDA) and European Medical Agency for the treatment of several solid tumor types, particularly those with PD-L1 expression or high microsatellite instability. In 2021, PD-1 blockade was approved by the FDA for patients with progressive metastatic solid tumors with a high tumor mutation burden (TMB-H; ≥10 mutations/Mb) who have no alternative treatment options (The ASCO Post) (1). The current study focused on lung cancer, which has the highest mortality rate among all cancers and a similar annual incidence in both men and women. Non-small cell lung cancer (NSCLC), which includes adenocarcinomas and squamous cell carcinoma (SCC), accounts for most lung cancer cases. Patients diagnosed with tumor stage III or IV NSCLC can undergo treatment with PD-1 blockade (2), and can have response rates of up to 30–45% within 9–18 months, with a durable response (>2 years) in some patients (3). However, the major challenge for patients, physicians, and the healthcare system is the lack of complete understanding of the mechanisms leading to progressive disease. Therefore, it is important to identify biomarkers of early clinical response during treatment, particularly for patients not likely to respond to immunotherapy.

The complexity of the immune system and the tumor microenvironment makes it unlikely that any single biomarker can predict the therapeutic response to PD-1 blockade in patients with NSCLC (4). While changes in circulating immune cell phenotypes and genetic markers after PD-1 blockade can correlate with clinical response, a limited number of studies have explored the potential of a combined genetic and immune cell phenotype as an early prognostic signature of clinical response in patients with NSCLC [reviewed in (5)]. In our study, the effects of PD-1 blockade on the tumor biology/genetics and the immune system in the same patient were explored based on the hypothesis that a combination of immunological and genetic biomarkers can improve the specificity of predicting clinical response to PD-1 blockade compared with either alone.

Results of clinical trials of PD-1 blockade have shown that the presence of tumor-infiltrating lymphocytes (TILs), and in particular, the number of CD8^+^ T cells, can be prognostic markers for clinical response for patients with NSCLC (6). A less invasive alternative to analysis of tumor biopises is longitudinal blood sampling for analysis of circulating immune cell subsets. Indeed, studies have shown that an increase in the frequency of CD8^+^ T cells in the blood, particularly with an activated phenotype or actively proliferating can identify patients with clinical benefit (7). Furthermore, clinical flow cytometric assays can be useful to characterize the functional status of the immune cells after PD-1 blockade, naïve, effector or memory T cells post-treatment compared to pre-treatment (8, 9). Such an assay would also satisfy the need for simple but comprehensive clinical tests to predict response to PD-1 blockade which are currently lacking.

Data derived from genetic profiling studies demonstrate that a subset of patients will clinically benefit from genome-driven oncology, thus a universal approach to next-generation sequencing (NGS)-based tumor profiling is important (10). Baseline TMB-H has been shown to be associated with clinical benefits in patients with NSCLC after PD-1 blockade (11). In patients with TMB-H tumors, the high expression levels of neoantigens recognized by activated CD8^+^ T cells after PD-1 blockade can result in the targeted killing of tumor cells, leading to a better response than in patients with low TMB (TMB-L) tumors (12, 13). However, not all studies have found a strong relationship between TMB status and durable response to immune therapy. The lack of technical guidelines regarding the calculation of TMB also makes the analysis more difficult and inconsistent. Apart from TMB analysis, the interpretation of mutations in individual tumor-driver or suppressor genes as pathogenic or likely pathogenic is also rapidly gaining importance for predicting the clinical response to PD-1 blockade in NSCLC. A favorable clinical benefit after PD-1 blockade has been reported for tumors bearing the gene of Kirsten rat sarcoma virus (*KRAS)* or combinations of *KRAS* and tumor protein P53 (*TP53*) mutations, independent of TMB-status or PD-L1 expression (14–16).

In this study, we report that changes in immune cell subsets occur early in the blood of patients with NSCLC, often within a period of 3–4 weeks after PD-1 blockade. Establishing a clinical assay that can distinguish both the phenotype and function of circulating immune cell subsets was beneficial to guide predictions of clinical response to PD-1 blockade. Furthermore, NGS of the tumor tissue of the same patients identified tumor-specific pathogenic or likely pathogenic mutations in Kelch-like ECH-associated protein 1 (*KEAP1*), neurogenic locus notch homolog protein 1 (*NOTCH1*), and *KRAS* only in responders. Multivariate analysis, combining immune and genetic parameters, identified a prognostic signature that could distinguish between responders and non-responders. After validation, the immune and genetic variables examined could be the basis for establishing new clinically relevant biomarkers of treatment response, analyzed in a clinical setting within 3–4 weeks after treatment initiation.

## 2 Materials and methods

### 2.1 Patients, study design, and sample collection

This was a prospective study of patients with stage III or IV NSCLC (n=15) diagnosed with adenocarcinoma, squamous cell carcinoma, or NSCLC NOS (not otherwise specified), recruited to the study before planned treatment start. Informed consent was obtained from the Regional Ethics Review Board in Gothenburg, Sweden (Permit number 953/18). Patients were recruited from April 2019 to April 2020, with a pause during the summer months of May to July (**Table 1**). The cohort included patients with single PD-1 (n=14) or PD-L1 (n=1) blockade as 1^st^ (n=10) or 2^nd^ (n=5) line therapy. Formalin-fixed paraffin-embedded (FFPE) tumor tissues were obtained from the cohort at the time of diagnosis (**Supplementary Figure 1A**). Participation in the study did not influence the course of the treatment or clinical procedures. Peripheral blood was collected from each patient in K2EDTA tubes at five-time points, before and after each consecutive 2–4-week treatment cycle, during routine clinical assessment (**Supplementary Figure 1A**). Blood was also collected in K2EDTA tubes on one occasion from healthy controls (n=3), who were age- and sex-matched with the patient cohort. PD-L1 staining of tumor biopsies before PD-1 blockade therapy was assessed according to routine clinical testing using PD-L1 28-8 antibody (Dako). The pathologist defined PD-L1 protein expression as the percentage of tumor cells exhibiting positive membrane staining at any intensity.

**Table 1:** Clinical characteristics of the NSCLC patients included in the study.

Patient cohort	n=15
Median age (range)	75 (65-82)
Sex, n (%)	
Male	11 (73)
Female	4 (27)
Smoking, n (%)	
Previous	10 (66)
Present	4 (27)
Never	1 (7)
Histology/diagnosis, n (%)	
Adenocarcinoma	8 (53)
Squamous cell carcinoma	5 (33)
NSCLC NOS (not otherwise specified)	2 (14)
PD-L1 status , n (%)	
>1%	1 (7)
1-50%	5 (33)
>50%	9 (67)
Stage at diagnosis, n (%)	
III	4 (17)
IV	11 (73)
Treatment, n (%)	
Prior chemoradiation	10 (66)
1st line immune therapy	5 (33)
PD-1 blockade	
Pembrolizumab (PD-1)	12 (80)
Nivolumab (PD-1)	2 (14)
Durvalumab (PD-L1)	1 (7)
Disease response at 9-10 months, n (%)	
Partial response	7 (46)
Complete response	1 (7)
Stable disease	2 (14)
Progressive disease	5 (33)

### 2.2 Clinical response

The clinical response to PD-1 blockade was determined every 3 months after obtaining the results of the CT-Scan, in line with the immune-related Response Evaluation Criteria in Solid Tumors (irRECIST) algorithm but assessed by an oncologist according to clinical judgment (17). The clinical response was divided into (1) complete response, no measurable tumor; (2) partial response, shrinkage in tumor size compared with baseline; (3) stable disease, no change in tumor size compared with baseline; and (4) progressive disease with increase in tumor size compared with baseline. In this study, responders were defined as patients who maintained a complete response, partial disease, or stable disease at 9 months (3^rd^ assessment) after the start of therapy to certify that they were indeed clinically responding to therapy. In one patient, the assessment was made 9-10 months post-treatment. Non-responders were those patients with a progressive disease before or at 9 months (**Table 1**).

### 2.3 Flow cytometry

Fresh whole blood in K2EDTA was stored at 22-24^0^C for 12–18 hours before FACS staining and analysis. Blood samples (1ml) were stained with antibody mix (**Supplementary Table 1**) and incubated for 20 minutes at 4^0^C. After lysis of red blood cells with a lysing solution at room temperature and two rounds of centrifugation and washing of the cells using a lyse wash assistant (Becton Dickinson, BD), the cells were acquired using a BD FACSCanto analyzer. Data analysis was performed using the Diva (BD) software based on the standardized phenotyping of human immune cells (9). Lymphocytes were enumerated using BD Multitest™ 6-color TBNK reagent (BD Biosciences) according to the manufacturer’s instructions.

### 2.4 Isolation and sequencing of tumor DNA

Genomic DNA was extracted from paired blood samples and FFPE tumor biopsies collected from the same patient. DNA isolated from the blood was used as a genomic DNA control for tumor tissue. Tumor tissue biopsies from 14 patients (**Table 2**) were included in the genetic analysis. The NGS panel used included 597 genes (Oncopanel All in One v2.8, Eurofins Genomics (Europe Sequencing GmbH, Germany), and the design covered 10 bp flanking regions of all exons (**Supplementary Table 2**). All steps from extraction, quantification, library preparation (Agilent Technologies Santa Clara, CA, USA), and sequencing were performed at Eurofins Genomics using optimized in-house protocols. Sequencing was performed on the Illumina NovaSeq 6000 platform (Illumina, San Diego, CA, USA) using 2 × 150 bp paired-end reads.

Table 2Summary of the clinical features and molecular pathogenic/likely pathogenic variants or VUS in cancer genes of responders and nonresponders.

**Table:** 

	Responders									
Clinical response*	SD	PR	CR	PR	PR	PR	SD	PR	PR	PR
**Patient coded ID**	1	2	3	4	5	6	7	8	9	10
**Tumor Stage**	IV	IV	III	IV	IV	IV	III	IV	IV	III
**Diagnosis**	NSCLC NOS	LUAD^#^	LUAD	LUAD	SCC^¶^	LUAD	LUAD	LUAD	LUAD	SCC
**Smoking**	Yes	Yes	Yes	Yes	Yes	Yes	Yes	Yes	Yes	Never
**PD-L1 expression**	<1%	>50%	>50%	>50%	>50%	1-49%	>50%	>50%	1-49%	>50%
**TMB (Mutations/Mb)**	33	4	2	2	0.7	35	11	8	3	22
**Variant analysis**										** **
***TP53***	Two Missense	Missense	None	None	Indel†	Inframe	Missense	Missense	None	Splice
***KRAS***	None	Missense	Missense	Missense	None	Missense	None	None	Missense	None
***STK11***	None	None	None	None	None	None	None	None	Indel†	None
***KEAP1***	None	None	None	None	None	Missense	Missense	None	None	None
***NOTCH 1***	None	None	None	None	None	None	Missense VUS	Missense VUS	None	Nonsense, Missense VUS
**Gene score**	2	2	1	1	1	3	3	2	2	3

**Table:** 

		Non-responders			
Clinical response*	PD	PD	PD	PD	PD
**Patient coded ID**	11	12	13	14	15
**Tumor Stage**	III	IV	IV	IV	IV
**Diagnosis**	NSCLC NOS	SCC	SCC	SCC	LUAD
**Smoking or VUS**	Yes	Yes	Yes	Yes	Yes
**PD-L1-expression**	1-49%	1-49%	>50%	1-49%	>50%
**TMB (Mutations/Mb)**	15	15	4	ND	1
**Variant analysis**					
***TP53***	None	Nonsense	Frameshift	ND	Missense
***KRAS***	None	None	None	None^a^	None
***STK11***	None	None	None	ND	None
***KEAP1***	None	None	None	ND	None
***NOTCH 1***	None	None	None	ND	None
**Gene score**	0	1	1	ND	1

*SD, Stable disease; PR, Partial response; CR, Complete response and PD, Progressive disease.

^#^Lung Adenocarcinoma ^¶^Squamous cell carcinoma.

^†^Insertion/Deletion.

a Based on clinical NGS data.

ND, Not determined.

### 2.5 Bioinformatics pipeline and interpretation analysis

Data quality assessment, mapping, and variant calling were performed using Eurofins Genomics in-house pipeline (Europe Sequencing GMB, Germany). Variant filtration was performed using Alissa Interpret software (Agilent Technologies, Santa Clara, CA, USA). A cutoff of 5% presence of the mutational allele was used for all filtrations. Alamut Visual (version 2.15; Sophia Genetics, Lausanne, Switzerland) and cBioportal (18, 19) were used to interpret variants. Mutations were further classified as a benign, likely benign, variant of unknown significance (VUS), likely pathogenic, or pathogenic, using the model described by Froyen et al. (20) and in accordance with ACMG and AMP guidelines. For mutational signature analysis, FastQC (version 0.11.2) was used to assess the quality of the data, and samtools (version 1.9) were used to sort, index, and assess mapping statistics. Paired-end reads were aligned to the human reference genome (hg19) using Burrows-Wheeler Aligner (BWA mem version, BWA_0.7.13) (21). Picard (version 2.2.4) was used to remove duplicates. The Genome Analysis ToolKit (GATK, version 4.1.3.0) (22) was used for base quality score recalibration, Mutect2 was used for calling and filtering somatic variants, and SigProfiler Extractor was used to extract mutational signatures (23). An analysis of variants in a selection of genes classified as pathogenic, likely pathogenic, or VUS in *TP53, KRAS*, serine/threonine kinase 11 (*STK11*)*, KEAP1*, and *NOTCH1* was combined into a gene score for each patient, where 0 indicates no variants, gene score of 1 indicates a single pathogenic/likely pathogenic variant or VUS in one gene, gene score of 2 indicates two pathogenic/likely pathogenic variants or VUS in one gene or a pathogenic/likely pathogenic variant or VUS in two genes, and a gene score of 3 indicates three pathogenic/likely pathogenic variants or VUS in one gene or a pathogenic/likely pathogenic variant or VUS in three different genes (**Table 2**).

### 2.6 TMB analysis

TMB was calculated using Eurofins Genomics by dividing the number of mutations by the size of the targeted coding region in megabases (Mb). Only non-synonymous missense variants were included in the calculation. The calculation was performed using the following exclusion criteria: non-coding mutations, mutations listed as known somatic mutations (according to cosmic v71), known germline mutations (in dbSNP), mutations with depth below 50x and allele frequency below 0.05, germline mutations with more than two counts in genome AD mutations (https://gnomad.broadinstitute.org/) in tumor suppressor genes (24, 25). Patients with a TMB of 10 mutations/Mb or higher are referred to as TMB-H (**Table 2**).

### 2.7 Multivariate analysis

Principal component analysis (PCA) was performed using the pca3d package in R. To examine the relationship between clinical responder or nonresponder (Y-variable) and the immune and tumor genetic signatures of the patients studied (X-variables), orthogonal partial least squares discriminant analysis (OPLS-DA) was performed using the SIMCA-P+ software (Sartorius GmbH, Göttingen, Germany). The quality of the OPLS-DA was based on the parameter R2Y, that is, the model’s goodness of fit (values ≥0.5, which define good discrimination and best possible fit, R2Y=1), and Q2, the goodness of prediction of the model (26). A Q2 value >0.4 is considered satisfactory with biological variables. Furthermore, the difference between the Q2 and R2Y values should not exceed 0.4. A combination of variable influence on projection (VIP) and VIPcvSE was used to exclude variables that were less likely to contribute to building the model. VIPcvSE is the confidence interval of the VIP. The significance of the separation between the groups in the OPLS-DA was calculated using CV-ANOVA (26).

### 2.8 Statistics

Non-parametric Mann–Whitney *U*-tests or Wilcoxon matched-pair signed rank tests were performed for unpaired and paired analyses, respectively, using the GraphPad Prism software (GraphPad Software, San Diego, USA). Progression-free survival (PFS) was estimated using the Kaplan–Meier method. The log-rank test was used to assess the differences in overall survival and PFS between the groups. Statistical significance was set at p <0.05, and no adjustments were made for multiple comparisons. Data analysis was performed using IBM SPSS Statistics version 27 (IBM, New York, USA) and GraphPad Prism software.

## 3 Results

### 3.1 Baseline frequencies of circulating B cells, but not bulk T cells, were associated with clinical response to PD-1 blockade

**Figure 1:**
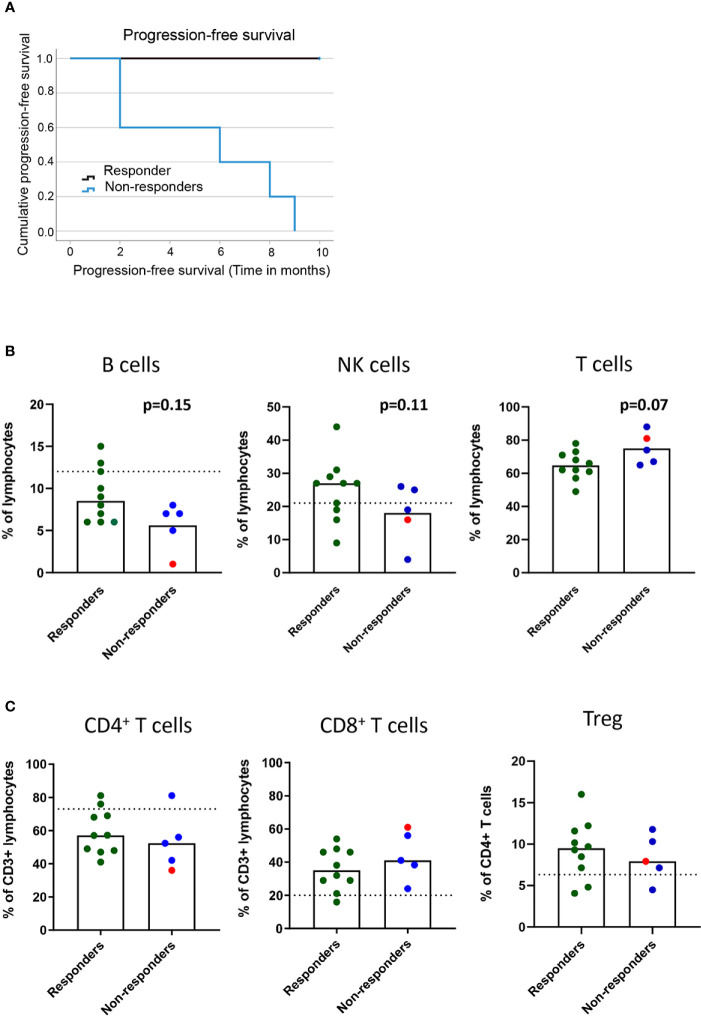
Baseline frequencies of circulating B cells, NK cells and T cells in association with the clinical response to PD-1 blockade. NSCLC patients received PD-1 blockade at 2-3 week cycles and blood was drawn at baseline for later flow cytometry analysis. **(A)** Kaplan-Meier curves of progression-free survival in responders and non-responders at the 9 months cutoff. **(B)** Frequencies of B cells, NK cells and CD3^+^ T cells **(C)** Frequencies of CD4^+^ and CD8^+^ T cells and regulatory T cells with memory phenotype (CD3^+^CD4^+^CD25^high^CD127^low^CD45RO^+^CD194^+^) in responder and non-responder patients as defined in the materials and methods section. Patient treated with anti-PD-L1 marked with red symbols. Dotted lines represent median frequencies of respective immune cell subsets for n=3 healthy individuals analyzed on one occasion.

In this patient cohort, the clinical assessment at the 9-10-month follow-up was based on iRECIST criteria. Responders (n=10) included one patient with a complete response, six patients with partial response and three patients with stable disease (**Table 1** and **Figure 1A**). Non-responders included patients with progressive disease observed at 3 (n=2), 6 (n=2), or 9 (n=1) months post-treatment (**Figure 1A**).

The events leading to clinical response or progression after PD-1 blockade therapy are multi-factorial, and the immune status before treatment initiation could impact the subsequent clinical response. We first investigated the baseline frequencies and fold-change after treatment of NK and B cells in our patient cohort (**Supplementary Figure 1B**). Although we could not quantify any significant change in the frequencies of circulating B cells (CD19^+^) or NK cells (CD3^-^CD56^+^) in the blood before and after treatment (*data not shown*), we found a trend for a higher baseline frequency of B cells (p=0.15) and NK cells (p=0.11) in responders than in non-responders (**Figure 1B**). The analysis of the frequencies of CD3^+^ T cells at baseline revealed a trend for higher frequencies of T cells in non-responders (p=0.07) than in responders (**Figure 1B**). However, no significant change was observed in the frequencies of CD4^+^ and CD8^+^ T cells in the blood before and after the 1^st^ cycle of treatment (*data not shown*) or in the baseline frequencies of CD4^+^ T cells and CD8^+^ T cells between responders and non-responders (**Figure 1C**). Finally, to test the hypothesis that increased regulatory T cell (Treg) frequencies correlate with poor prognosis, we analyzed the frequencies of CD4^+^CD25^+^CD127^-^CD45RO^+^CCR4^+^ Tregs in circulation (**Supplementary Figure 1B**). We did not observe any difference in baseline levels of Tregs between responders and non-responders (**Figure 1C**), nor any change that could be associated with clinical response after the 1^st^ cycle of treatment (*data not shown*).

In summary, while frequencies of the bulk CD4^+^ and CD8^+^ populations could not predict treatment response, a trend for higher baseline frequencies of B cells and NK cells in responders and CD3^+^ T cells in non-responders was observed after PD-1 blockade in our cohort of patients.

### 3.2 Elevated frequencies of activated effector memory CD4^+^ and CD8^+^ T cells at early post-treatment time points in patients responding to PD-1 blockade

**Figure 2:**
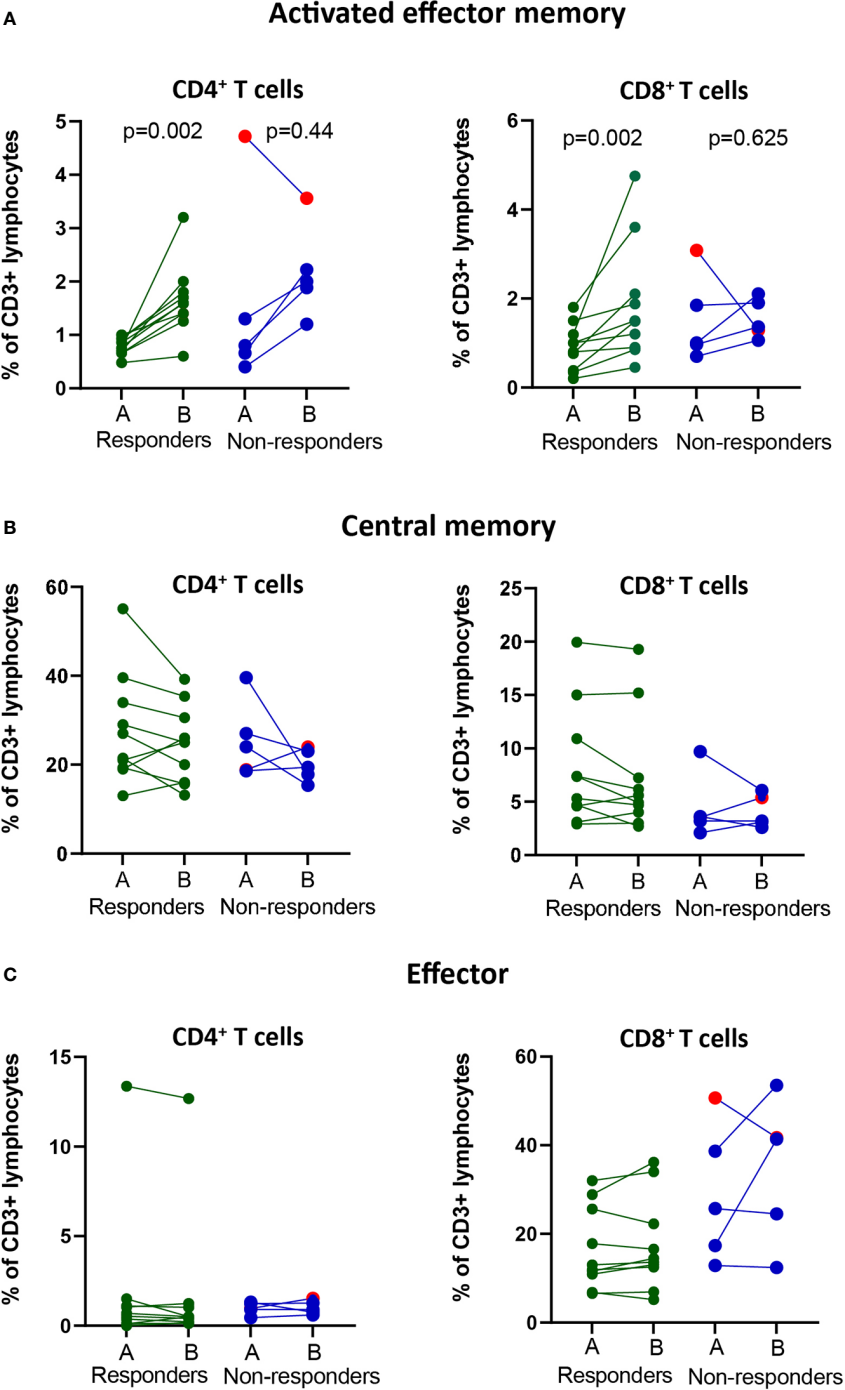
Changes in frequencies of effector and memory T cell populations in the blood of responders and non-responders after PD-1 blockade. NSCLC patients received PD-1 blockade at 2-3 week cycles and blood was drawn at pre-(A) compared post-1^st^ treatment cycle before for later flow cytometry analysis. **(A)** The frequencies of activated effector memory T cells (CD3^+^CD4^+^/CD8^+^CD45RA^-^CCR7^-^CD38^+^HLA-DR^+^) in circulation of responders and non-responders. **(B)** The frequencies of central memory T cells (CD3^+^CD4^+^CD8^+^CD45RA^-^CCR7^+^) in circulation of responders and non-responders. **(C)** The frequencies of effector T cells (CD3^+^CD4^+^/CD8^+^CD45RA^int^CCR7^-^) in circulation of responders and non-responders. Patient treated with anti-PD-L1 marked with red symbols.

Since the frequencies of bulk T cell populations did not change after treatment, we continued to analyze specific subsets of effector or memory T cells using a clinical assay based on the expression of CD45RA and CCR7 markers and activation markers CD38 and HLA-DR (9) (**Supplementary Figure 1B**). To determine the time at which changes in the frequencies of the immune cell subsets were observed after treatment, we analyzed pre-treatment and four cycles post-treatment (A-E) fresh whole blood samples. Our analysis revealed that for all patients except one with progressive disease, there was an increase in the frequencies of activated effector memory CD4^+^ and CD8^+^ T cells after the 1^st^ or 2^nd^ treatment cycle, followed by a decrease at the 3^rd^ or 4^th^ treatment cycle, reaching either pre-treatment levels or somewhat higher levels (**Supplementary Figure 1C**). In the responder group, an increase (p<0.01) in the frequency of both CD4^+^ and CD8^+^ activated effector memory (CD45RA^-^CCR7^-^CD38^+^HLA-DR^+^) T cells was observed after the 1^st^ cycle of treatment compared with the pre-treatment levels (**Figures 2A**). When analyzing the fold-change between pre- and post treatment values, a significant difference between responders and non-responders (p=0.048) was only observed for the activated effector memory CD8^+^ T cell subset (**Supplementary Figure 2A**).

Analysis of central memory (CD45RA^-^CCR7^+^) CD4^+^ and CD8^+^ T cells for all treatment cycles revealed a decrease in frequency after the 1^st^ treatment cycle in 6/10 patients in the responder group and 2/5 patients in the non-responder group (**Supplementary Figure 1D**). The frequencies of central memory cells increased over time in individual patients and reached pre-treatment frequencies at the 4^th^ treatment cycle for most patients, independent of clinical response (**Supplementary Figure 1D**). The trend for a decrease in the frequency of central memory CD4^+^ and CD8^+^ T cells at 1^st^ cycle after treatment compared to pre-treatment was however not significant (**Figure 2B**). Further, we observed no fold-change differences in the frequencies of central memory CD4+ or CD8+ T cells between the responders and non-responders (**Supplementary Figure 2B**).

Finally, the analysis of effector (CD45RA^+^CCR7^neg^) CD4^+^ and CD8^+^ T cells did not reveal any changes in the pre- and post-treatment levels over time in either the responder or the nonresponder group (**Supplementary Figures 1E**). Interestingly, a high frequency of CD4^+^ effector T cells was observed in one patient with a clinically durable response (>2 years) (**Figure 2C**). The fold-change difference in the frequencies of effector T cells pre- and post-treatment could not distinguish the reponders from non-responders (**Supplementary Figure 2C**).

In summary, in our cohort of patients, a post-treatment significant increase in the frequency of CD8^+^ activated effector memory cells could indicate clinical response to PD-1 blockade.

### 3.3 Tumor DNA sequencing analysis revealed that mutation in *KRAS* was associated with the response to PD-1 blockade

**Figure 3:**
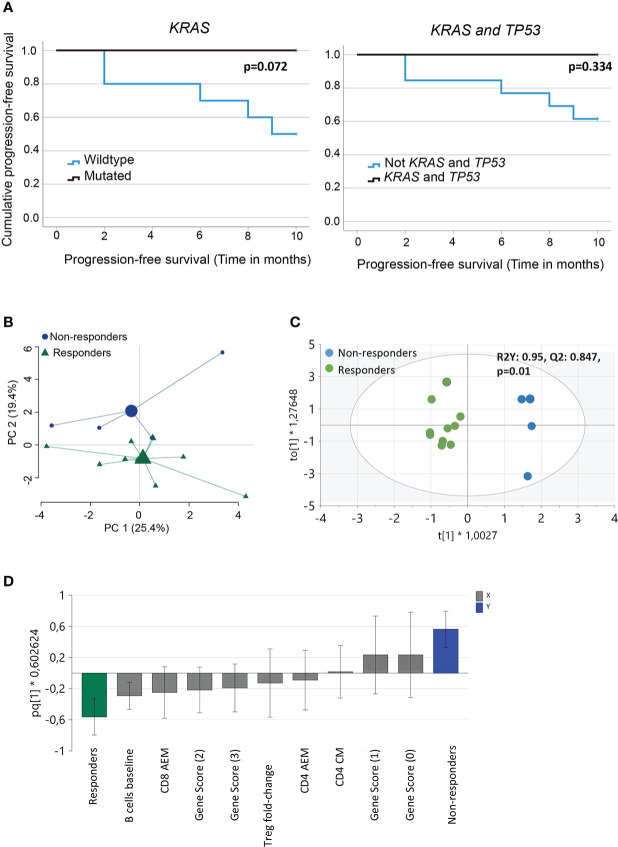
Analysis of immune and genetic variables measured and their relation to clinical outcome. **(A)** Kaplan-Meier curves estimates comparing overall survival of patients based on *KRAS* or *KRAS* and *TP53* mutational status of the tumor **(B)** Principal component analysis of variables measured (baseline frequencies of B cells, NK cells, CD3^+^ T cells, CD4^+^ and CD8^+^ T cells and Tregs, pre- and post- 1st cycle of treatment, fold-change activated effector memory T cells, central memory T cells, effector T cells and Tregs, gene score, PD-L1 status and TMB score). Large symbols, green (responder) and blue (non-responders) indicate weighted means of the groups **(C)** Score scatter plot and **(D)** loading column plot from an orthogonal partial least squares‐discriminant analysis (OPLS‐DA) of the X variables in **(B)**, using a VIP cutoff > 0.8 and VIPcvSE cutoff <1.5, and Y variables (responders/non-responders). R2Y defines the goodness of fit, and Q2 the goodness of prediction. Total number of patients for the analysis (n=14) with responders (n=10) and non-responders (n=4).

We performed NGS of isolated DNA from pre-treatment tumor biopsies to explore the potential of a combined genetic and immune cell signature as an early biomarker of clinical response to PD-1 blockade. We analyzed gene variants in tumor-specific genes, including *TP53, STK11, KRAS*, and *KEAP1* (**Supplementary Table 2**), and an in silico panel of 59 immune-related genes. The analysis revealed that *TP53* mutations were frequent, with missense, nonsense, in-frame, and splice mutations detected in 10/14 patients, independent of the clinical response. Our analysis also showed no significant difference in the time to progression at 9 months for patients with wild-type or mutated *TP53* (**Supplementary Figure 3A**). Furthermore, in our patient cohort, *KRAS* mutations (5/10 patients) or *KRAS* and *TP53* mutations were detected only in the responders (**Table 2**). Although published data (27) suggest that patients with mutations in *KRAS^mut^* or *TP53* treated with PD-1 blockade experience a longer PFS than patients with wildtype (WT) tumors, we observed only a trend in patients with *KRAS* ^mut^ tumors (p=0.072), possibly because of the low number of patients in this study cohort (**Figure 3A**).

Analysis of the 59-gene immune panel revealed truncating and missense variants in several genes. However, only one nonsense mutation in the tumor suppressor gene *NOTCH1* in responders was predicted to be likely pathogenic. Variants in the additional genes were detected but predicted to be VUS (**Supplementary Table 3**). The number of pathogenic, likely pathogenic, and VUS mutations detected in individual patients was the basis for a gene score of ≥ 2 in 7/10 patients in the responder group and ≤ 1 in 5/5 patients in the nonresponder group. This indicates that pathogenic and likely pathogenic mutations were more common in the responders (**Table 2** and **Supplementary Table 3**). We conclude that both the number of variants in genes and the pathogenicity of these variants could contribute to the clinical response. Thus, interpreting the pathogenicity of gene variants could be important to consider for future studies.

Finally, we investigated the relationship between TMB score, PDL-1 status, and clinical response in our cohort. The patients with TMB-H (>10 mutations/Mb) were distributed among both responders and non-responders (**Table 2**). Neither TMB-H nor PD-L1 expression (>1%) could be associated with differences in the time to PFS between responders and non-responders (**Supplementary Figure 3B**).

In summary, we found that driver mutations, particularly in *KRAS*, was related to clinical response after PD-1 blockade but not TMB score or PD-L1 expression.

### 3.4 Multivariate analysis can define the relationship between the measured immune and genetic parameters and clinical response to PD-1 blockade

Next, we performed multivariate analyses to test our hypothesis by analyzing the relationship between immune and genetic variables and their relationship to clinical response after PD-1 blockade. The baseline frequencies of B cells, NK cells, CD3 ^+^ T cells, CD4 ^+^ and CD8 ^+^ T cells, and Tregs were included in the analysis. In addition, we also added pre- and post- 1^st^ cycle of treatment, fold-change activated effector memory T cells, central memory T cells, effector T cells, and Tregs. The clinical and genetic parameters included the gene score, PD-L1 status, and TMB score. PCA, including all these variables, indicated a separation between responders and non-responders, although only about 45% of the variance was explained by the two principle components (**Figure 3B**). To further define the variables most important for discrimination between the groups, OPLS-DA was performed using a VIP cutoff >0.8 and a VIPcvSE cutoff <1.5. The analysis showed that the two clinical response groups, responders and non-responders, were separated with high discrimination and predictability (R2Y:0.95, Q2:0.847, P=0.01) (**Figure 3C**) based on the combination of immune and genetic variables. OPLS-DA analysis, including only immune or genetic variables alone, resulted in poor discrimination and predictability values (immune parameters: R2Y: 0.756; Q2: 0.389 and genetic parameters R2Y: 0.52; Q2: 0.119). The variables best defining the clinical responder group were baseline frequencies of B cells, fold-change activated effector memory CD8^+^ T cells, and gene score >1 (**Figure 3D**). In summary, we report that a combination of genetic and immune parameters analyzed before or within 3–4 weeks after the start of PD-1 blockade therapy could differentiate responders from non-responders.

## 4 Discussion

Cancer therapy targeting the PD-1/PD-L1 pathway of immune regulation has been approved as a first- or second-line therapy for a growing list of malignancies, including NSCLC. However, robust biomarkers of clinical response to PD-1 blockade are still lacking. Several studies have reported the potential of peripheral blood-based and tumor-based biomarkers, such as TMB score, gene signatures, PD-L1 expression, phenotypes of TILs, and multiplex immunohistochemistry assays for qualifying TILs (28–31). We aimed to identify prognostic biomarkers of clinical response to PD-1 blockade that are evident in fresh blood and archival tissue biopsies within the 1^st^ few weeks of treatment. We reported that a combination of genetic and immune-related variables measured in patients before and during the early stages of treatment could indicate a clinical response. When confirmed in a larger validation cohort, our findings can lead to the development of clinical tests for predicting early responses to PD-1 blockade.

Currently, immunohistochemical staining for PD-L1 in tumor biopsies is one of the approved predictive biomarker of clinical response that supports the choice of PD-1 blockade for patients with NSCLC. In our patient cohort, we observed, in accordance with published studies, that PD-L1 expression was variable among patients in both response groups. In addition, no difference in time to PFS at the 9–10 months cutoff was observed in patients with TMB-H (>10 mutations/Mb) or TMB-L (<10 mutations/Mb). Recently, the FDA approved anti-PD-1 (pembrolizumab) for a TMB score of >10 mutations/Mb based on the Foundation One^®^ clinical assay in patients with solid tumors. A TMB score of >10 could be a predictive biomarker of response to PD-1 blockade (32) and possibly be compared with PD-L-1 expression in tumor tissue. However, the foundation One^®^ clinical assay is expensive to use in all clinical settings, which led us to develop an in-house variant interpretation workflow (33). We investigated the mutations/variants in tumor-specific genes and further classified the variants based on their pathogenicity and effect, rather than only including all variants present in the tumor, which can be considered as a drawback of the TMB assay.

We first analyzed the activating mutations in *KRAS*, which is an estimated 35% of lung adenocarcinomas and is one of the most prevalent oncogenic drivers in NSCLC. However, patients with stage IV lung adenocarcinoma and *KRAS* ^mut^ seem to benefit from long-term response rates, particularly after first-line PD-1 blockade, compared with patients receiving platinum doublet treatment (34). An explanation for the preferential response to PD-1 blockade could be that *KRAS* ^mut^ tumors often express high levels of PD-L1 because of activation of the downstream p-ERK signaling pathway (35). Furthermore, when *KRAS* ^mut^ tumors are TMB-H, could through high neoantigen expression, lead to the activation of CD8^+^ T cells in the tumor tissue after PD-1 blockade. Altogether, these studies can explain why patients with *KRAS^mut^* tumors respond to PD-1 blockade (36). Further studies are warranted to address the mechanisms leading to the long-term clinical response in patients with *KRAS^mut^* tumors to further improve treatment and response to PD-1 blockade (37).

In addition to KRAS, the other most common mutation detected in the tumor tissue of patients is the tumor suppressor gene *TP53*. In line with previous reports, we found that *TP53* and *KRAS* mutations occur in responders (37). Thus, we believe that interrogating the KRAS-mutant status for patients with *TP53* mutations could potentially improve the prognostic differentiation of responders and non-responders. Furthermore, its advantage compared to large panels or whole exome sequencing needed for TMB, is that the analysis of a selected number of gene variants such as *KRAS, TP53, KEAP-1, STK-11*, and *NOTCH-1* would be possible with a small gene panel, which might sufficiently provide the necessary prognostic information in combination with the immune cell subset analysis described below.

The effects of PD-1 blockade on bulk immune cell subsets in melanoma and NSCLC patients have shown that the changes were evident early after treatment (7, 38, 39). In a report using RNA sequencing and the CYBERSORT technique to analyze immune cell populations in the blood of patients with NSCLC after the 1^st^ cycle of treatment, fewer CD8^+^ T cells were found before therapy in patients with durable clinical responses than in patients with progressive disease. However, a trend that our study and other studies similar to ours, have been unable to confirm (7, 40). It can be speculated that for patients with a durable clinical response, the low frequency of CD8^+^ T cells in the circulation pre-treatment indicates improved migration to the tumor tissue compared with CD8^+^ T cells from patients with progressive disease. Therefore, it would have been interesting to analyze the migration of CD8^+^ T cells to the tissue by comparing pre- and post-treatment tumor biopsies. However, due to the ethical restrictions of sampling biopsies from patients with lung cancer before and after therapy, the effect of PD-1 blockade on immune cell subsets in the tumor tissue is currently unknown and requires further investigation.

Another interesting finding of our study was the trend of elevated baseline frequency of CD19^+^ B cells in responders. B cells can contribute to tumor immunity by functioning as antigen-presenting cells, presenting tumor antigens to T cells in the tumor tissue. B cells can also be activated and differentiate into autoantibody-producing plasma cells, triggering autoimmunity, particularly in patients who receive a combination of CTLA-4 and PD-1 blockade (41, 42). Furthermore, B cells can form tertiary lymphoid structures in tumor tissue, which indicates a positive clinical response to PD-1 blockade. (43). Further studies will shed light on the phenotype, location, and possible expansion of specific subsets of B cells, which can be related to their function and clinical response.

Although previous studies in patients with NSCLC have revealed specific immune cell populations related to tumor immunity after PD-1 blockade, none have addressed the usefulness of a clinical test that could address both the phenotypic and functional characteristics of circulating immune cells (8, 9). We detected changes in the frequency of both CD4^+^ and CD8^+^ activated effector memory and central memory cells but not effector T cells. A unique feature of our study is the analysis of a combination of CD38 and HLA-DR to identify activated effector memory T cells previously reported in the context of HIV infection (44). An increase in activated effector memory CD8^+^ T cells post-treatment compared with pre-treatment was detected in all but one patient. However, the difference in fold-change pre- compared to post-treatment was significant between responder and non-responders, although these findings should be validated in a larger cohort of patients. In a recent study of patients with metastatic melanoma, PD-1 blockade was associated with an increase in the frequency of activated effector memory CD8^+^ T cells in the blood, as revealed by single-cell RNA sequencing after the 2^nd^ cycle of treatment, and only in responders, strengthening the observations in our study (39).

The PCA analysis indicated a discrimination between the groups, although only approximately 45% of the variance was explained by the two principle components which is considered low. This limitation can most likely be explained by the low number of patients together with the selection of variables. OPLS discriminant analysis with VIP selection was used to select variables most important for discrimination between the groups which narrowed down the variables measured and revealed that an increase in the frequency of activated effector memory CD8^+^T cells after treatment is one of the important factors, together with baseline frequencies of B cells, to determine the clinical response to PD-1 blockade. Importantly, the combined immune and genetic parameters was associated with the clinical response to PD-1 blockade in this cohort of patients with NSCLC. The study’s main strengths include well-characterized patients, longitudinal analysis of patients, and classification of variants based on pathogenicity, which was a salient feature of our study. The classification of the variants will guide the selection of variants that are important co-mutations in each patient and will also strengthen the multivariate analysis. However, the small number of patients is a limitation to the generalizability of the study outcomes and might have influenced the PCA, the fit and predictive ability of the OPLS-DA model. Therefore, validation studies, including more study sites and patients, is important and ongoing.

## 5 Conclusions

In conclusion, we propose that detecting changes in immune cell subsets can predict clinical outcomes when combined with other parameters, such as pathogenic, likely pathogenic, or VUS mutations in specific genes, including *KRAS, KEAP-1, STK-11, NOTCH-1*, and *TP53*. Our study is one of the few prospective studies in which pre-treatment analysis of immune cell subsets in the blood is compared with post-treatment cycles using flow cytometry and NGS of the tumor tissue from the same patient to predict the clinical response to PD-1 blockade. We argue that the mechanism underlying the response to PD-1 blockade in patients with NSCLC is multi-factorial and cannot be based on PD-L1 or TMB scores alone. The results of this pilot study, when validated in a larger cohort of patients, could be useful in monitoring clinical response within 3–4 weeks after PD-1 blockade.

## Data availability statement

The raw data supporting the conclusions of this article will be made available by the authors, without undue reservation.

## Ethics statement

The studies involving human participants were reviewed and approved by Regional Ethics Review Board in Gothenburg, Sweden. The patients/participants provided their written informed consent to participate in this study.

## Author contributions

SR, AR and AH conceptualized the study. AH, EE, LA and PT collected the clinical samples and interpreted the clinical data. ND, AR, SR, FN, AL, EE and MM acquired or analyzed the data for the generation of figures. ND, AR, SR and AH wrote the manuscript with major contribution from VS, MM, EE and AL. All authors contributed to the article and approved the submitted version.

## Supplementary material

Supplementary Figure 1 Time course analysis of frequencies of activated effector memory, central memory T cells and effector T cells in the blood of NSCLC patients, pre- and post-PD-1 blockade. **(A)** NSCLC patients were recruited to the study and tumor tissue was analyzed for mutation status before the start of PD-1 blockade. Blood was drawn before treatment and at 2–3-week cycles (maximum 4-cycles) for flow cytometry analysis. Clinical response cutoff to calculate PFS was 9-10 months. **(B)** Gating strategy for the analysis frequencies of B cells (CD19), NK cells (CD16/56), regulatory T cells with memory phenotype (CD3^+^CD4^+^CD25^high^CD127^low^CD45RO^+^CD194^+^ (CCR4)) and CD4^+^ and CD8^+^ T cells based on the expression of CD45RA and CD197 (CCR7), further classified into Naïve (I), Central memory (II), Effector memory (III) or Effector (IV) CD4^+^ and CD8^+^ T cell subsets. The expression of activation molecules CD38 and HLA-DR was studied on the effector memory population (III) **(C)** The frequencies of activated effector memory T cells (CD3^+^CD4^+^/CD8^+^CD45RA^-^CCR7^-^CD38^+^HLA-DR^+^) in circulation of patients pre- and post-treatment cycle for responders and non-responder patients. **(D)** The frequencies of central memory T cells (CD3^+^CD4^+^/CD8^+^CD45RA^-^CCR7^+^) in circulation of patients pre- and post-treatment cycle for responders and non-responder patients. **(E)** The frequencies of effector T cells (CD3^+^CD4^+^/CD8^+^CD45RA^int^CCR7^-^) in circulation pre- and post-treatment cycle for responders and non-responder NSCLC patients. Patient treated with anti-PD-L1 marked with red symbols.

Supplementary Figure 2 Fold-change post-treatment compared to baseline in the frequencies of activated effector memory, central memory and effector T cells in the blood of NSCLC patients. **(A)** Fold-change in frequencies of activated effector memory T cells (CD3^+^CD4^+^/CD8^+^CD45RA^-^CCR7^-^CD38^+^HLA-DR^+^) cells in circulation of responders and non-responders. **(B)** Fold-change in frequencies of central memory T cells (CD3^+^CD4^+^CD8^+^CD45RA^-^CCR7^+^) cells in circulation of responders and non-responders. **(C)** Fold-change in frequencies of effector T cells (CD3^+^CD4^+^/CD8^+^CD45RA^int^CCR7^-^) in circulation of responders and non-responders. Patient treated with anti-PD-L1 marked with red symbols.

Supplementary Figure 3 Kaplan-Meier curves to estimate progression free survival of patients at the 10 months cutoff stratified on PD-L1 expression and tumor mutational burden. **(A)** Kaplan-Meier estimates comparing progression free survival of patients based on mutation in tumor suppressor *TP53.* **(B)** Kaplan-Meier estimates comparing progression free survival of patients based on TMB-H or TMB-L **(C)** Kaplan-Meier estimates comparing progression free survival of patients based on PD-L1>1% PD-L1<1-49% and PD-L1>50%.
