# Supplementary material for: Combinatory analysis of immune cell subsets and tumor-specific genetic variants predict clinical response to PD-1 blockade in patients with non-small cell lung cancer
Source: Front Oncol. 2023 Feb 9;12:1073457. doi: 10.3389/fonc.2022.1073457 (PMC9948027; doi:10.3389/fonc.2022.1073457)
Supplement: Supplementary Figure 1 — Time course analysis of frequencies of activated effector memory, central memory T cells and effector T cells in the blood of NSCLC patients, pre- and post-PD-1 blockade. (A) NSCLC patients were recruited to the study and tumor tissue was analyzed for mutation status before the start of PD-1 blockade. Blood was drawn before treatment and at 2–3-week cycles (maximum 4-cycles) for flow cytometry analysis. Clinical response cutoff to calculate PFS was 9-10 months. (B) Gating strategy for the analysis frequencies of B cells (CD19), NK cells (CD16/56), regulatory T cells with memory phenotype (CD3+CD4+CD25highCD127lowCD45RO+CD194+ (CCR4)) and CD4+ and CD8+ T cells based on the expression of CD45RA and CD197 (CCR7), further classified into Naïve (I), Central memory (II), Effector memory (III) or Effector (IV) CD4+ and CD8+ T cell subsets. The expression of activation molecules CD38 and HLA-DR was studied on the effector memory population (III) (C) The frequencies of activated effector memory T cells (CD3+CD4+/CD8+CD45RA-CCR7-CD38+HLA-DR+) in circulation of patients pre- and post-treatment cycle for responders and non-responder patients. (D) The frequencies of central memory T cells (CD3+CD4+/CD8+CD45RA-CCR7+) in circulation of patients pre- and post-treatment cycle for responders and non-responder patients. (E) The frequencies of effector T cells (CD3+CD4+/CD8+CD45RAintCCR7-) in circulation pre- and post-treatment cycle for responders and non-responder NSCLC patients. Patient treated with anti-PD-L1 marked with red symbols. [file DataSheet_1.pdf]

Supplementary Data:

Combinatory analysis of immune cell subsets and tumor-specific genetic variants can predict clinical response to PD-1 blockade for non-small cell lung cancer patients

Dutta et al.

Supplementary Figure 1, 2 and 3

Supplementary Table 1, 2 and 3

SUPPLEMENTARY FIGURE 1

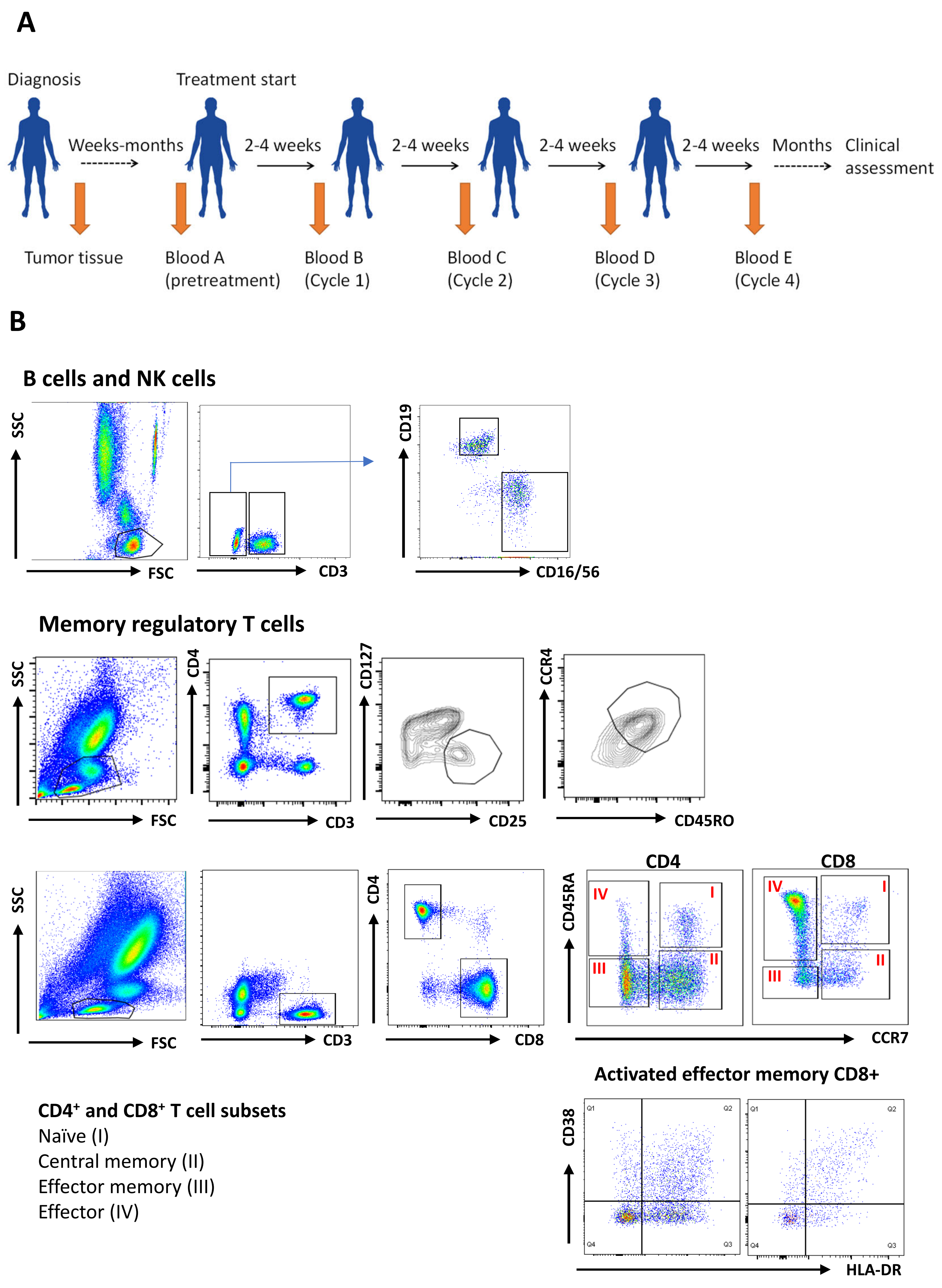

C

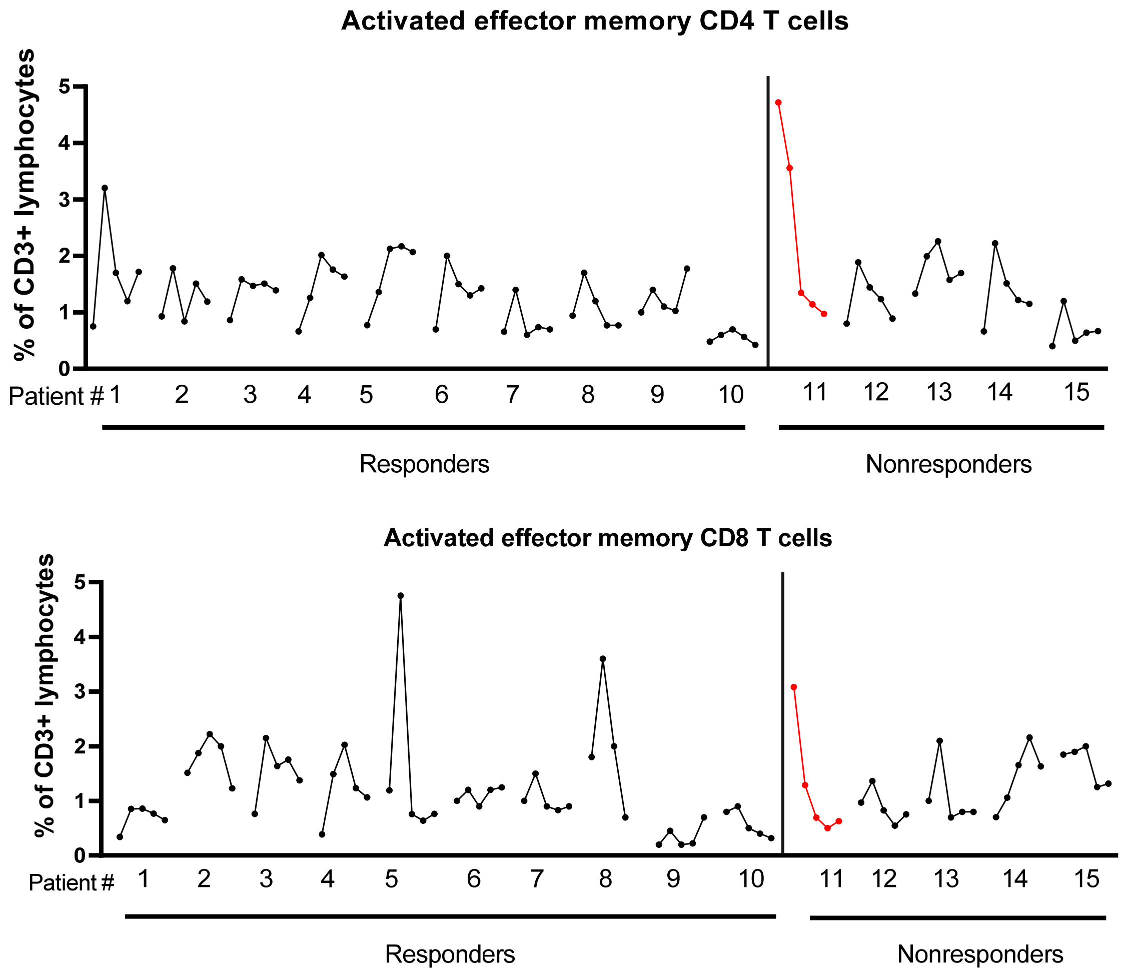
Activated effector memory CD4 T cells
% of CD3+ lymphocytes
0
1
2
3
4
5
Patient # 1 2 3 4 5 6 7 8 9 10 11 12 13 14 15
Responders
Nonresponders
Activated effector memory CD8 T cells
% of CD3+ lymphocytes
0
1
2
3
4
5
Patient # 1 2 3 4 5 6 7 8 9 10 11 12 13 14 15
Responders
Nonresponders

D

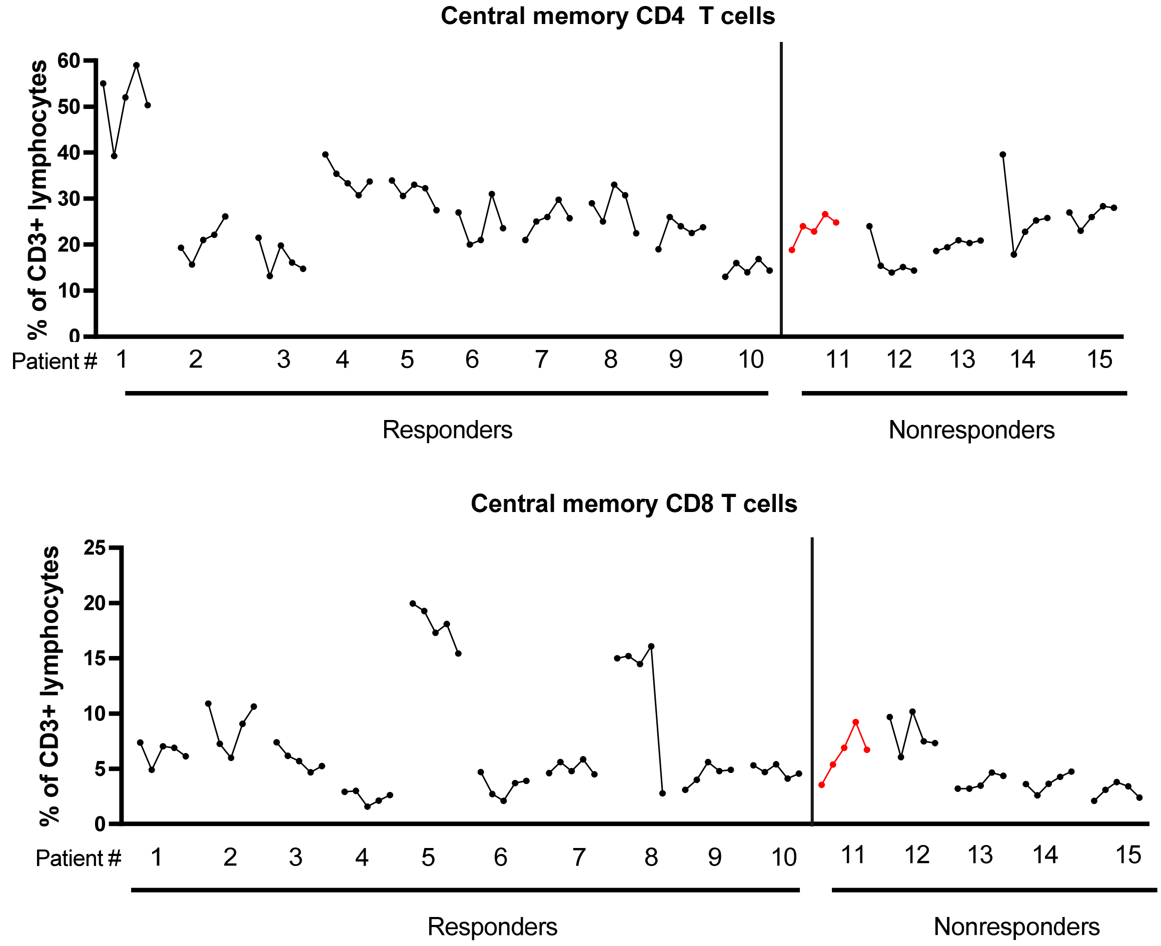
Central memory CD4 T cells
% of CD3+ lymphocytes
0
10
20
30
40
50
60
Patient # 1 2 3 4 5 6 7 8 9 10 11 12 13 14 15
Responders
Nonresponders
Central memory CD8 T cells
% of CD3+ lymphocytes
0
5
10
15
20
25
Patient # 1 2 3 4 5 6 7 8 9 10 11 12 13 14 15
Responders
Nonresponders

E

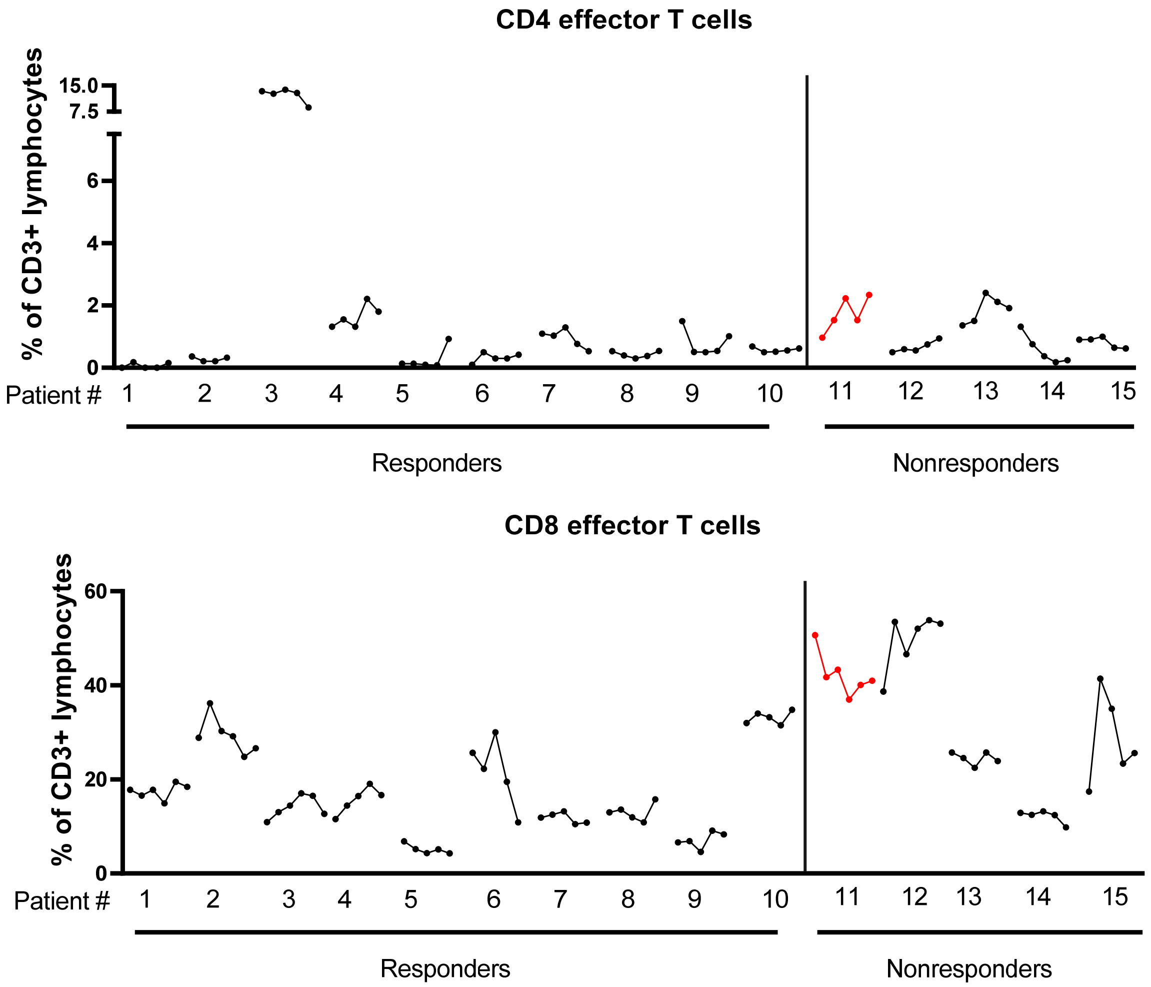
CD4 effector T cells
% of CD3+ lymphocytes
15.0
7.5
6
4
2
0
Patient #
1
2
3
4
5
6
7
8
9
10
11
12
13
14
15
Responders
Nonresponders
CD8 effector T cells
% of CD3+ lymphocytes
60
40
20
0
Patient #
1
2
3
4
5
6
7
8
9
10
11
12
13
14
15
Responders
Nonresponders

SUPPLEMENTARY FIGURE 2

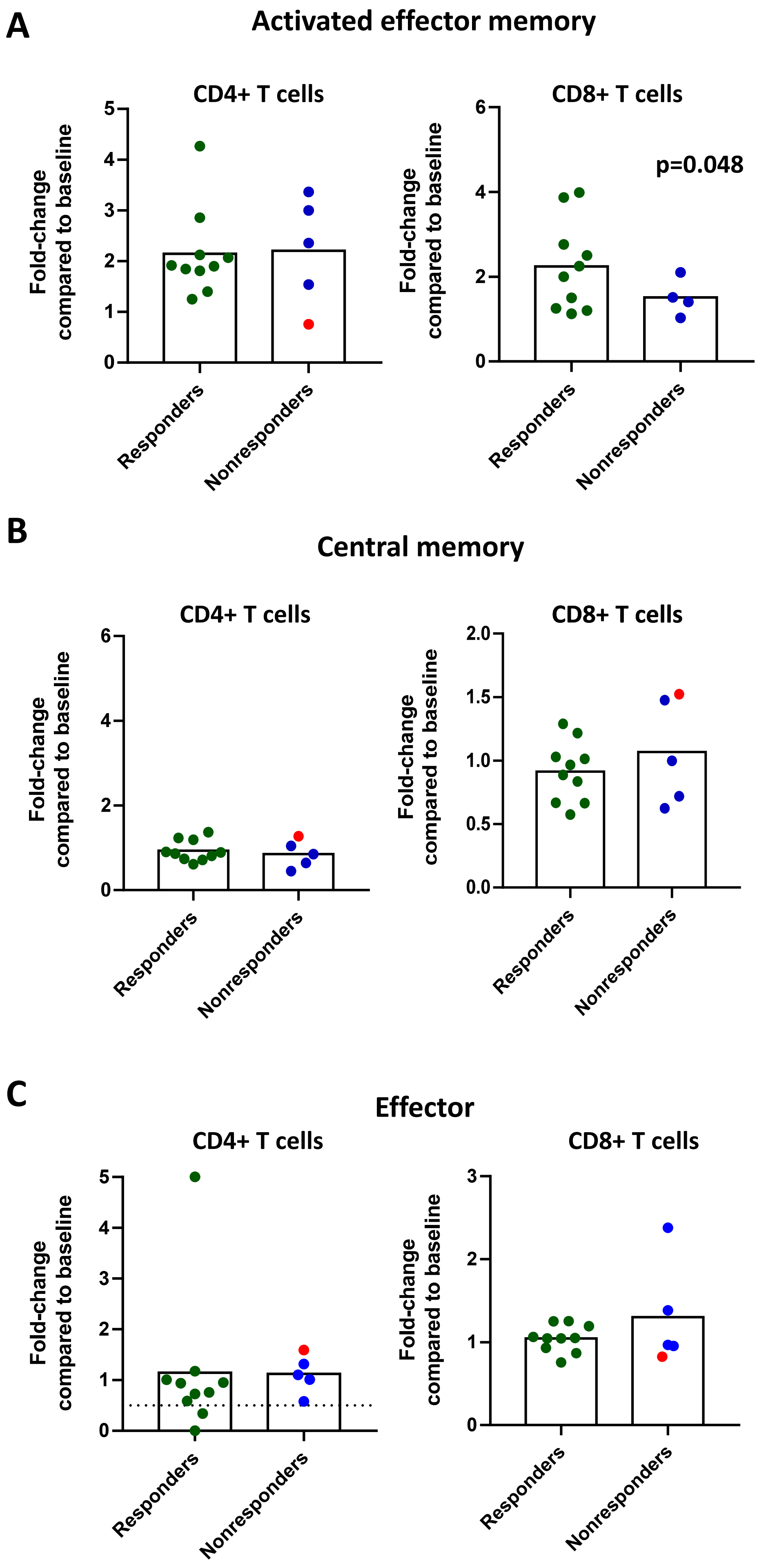

SUPPLEMENTARY FIGURE 3

A

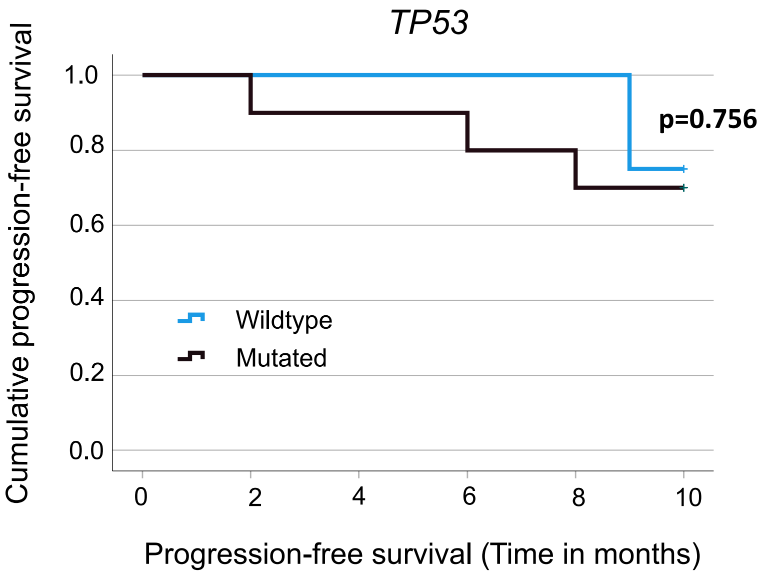

B

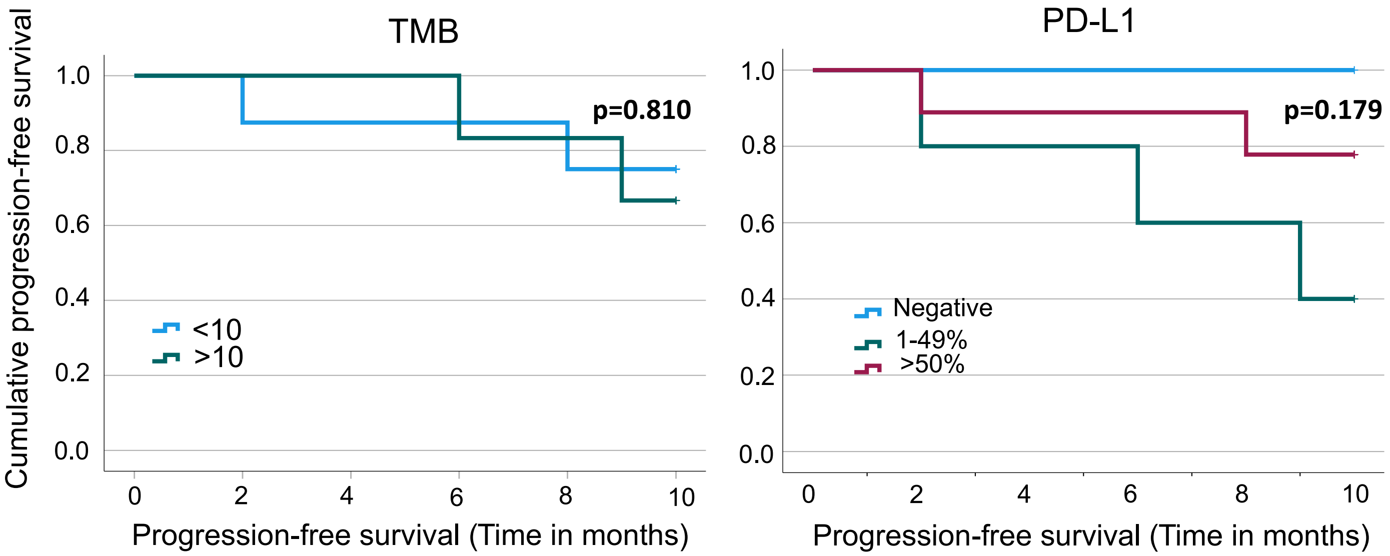

Supplementary Table 1

Antibodies for flow cytometry staining.

**Antibodies and clone**	**Vendor**
CD31 (WM 59)	BD Biosciences
CCR7 CD197 (2-L1-A)	BD Biosciences
CD4 (SK3)	BD Biosciences
CD45RA (HI100)	BD Biosciences
CD38 (HIT2)	BD Biosciences
CD8 (SK-3)	BD Biosciences
CD3 (UCHT1)	BD Biosciences
HLA-DR (G46-6)	BD Biosciences
CD25 (M-A251)	BD Biosciences
CCR4 CD194 (1G1)	BD Biosciences
CD127 (HIL-7R-M21)	BD Biosciences
CD45RO (UCHL1)	BD Biosciences

Supplementary Table 2

Genes included in gene panel analysis (in-silico).

Immune genes:

B2M
CD274
CD74
CEBPA
CSF1
TENT5C
FAS
FLT3
GATA1
GATA2
GATA3
GATA4
GATA6
GREM1
IL2RA
IL2RB
IL2RG
IL6
IL6ST
IL7R
IRAK4
IRF2
IRF4
JAK1
KIT
LCK
NFKBIA
NOTCH1
NOTCH2
NOTCH3
PRF1
RARA
RARB
RARG
S1PR2
SMAD2
SMAD3
SMAD4
SMARCA4
SMARCB1
SMARCE1
SOCS1
STAT3
STAT4
TNFAIP3
TNFRSF14
VEGFA
CBLB
FOXP1
IGF1R
IKZF1
IKZF4
PCGF2
PDCD1LG2
TYK2
POT1
SMO
JAK2
JAK3

Cancer genes

TP53
KRAS
STK11
KEAP1

Supplementary Table 3

DNA variants detected in the tumor biopsies of responder and non-responder patients

Patient	**DNA-variant (allele frequency %)**	**Interpretation of DNA variant**
Responders		
1	NM_000435.3(***NOTCH3***):c.260G>T, p.(Cys87Phe) 9.8%	VUS
	NM_000546.5(***TP53***):c.375G>C , p.Thr125=(19.7%)	likely pathogenic
	NM_000546.5(***TP53***):c.843C>A , p.Asp281Glu (12.8%)	likley pathogenic
2	NM_033360.4(***KRAS***):c.34G>T, p.(Gly12Cys) (36.2%)	pathogenic
	NM_000546.5(***TP53***):c.454C>T, p.(Pro152Ser) (29.1%)	likely pathogenic
	NM_000222.2(***KIT***):c.1684G>C, p.(Glu562Gln) 22%	VUS
3	NM_033360.4(KRAS):c.34G>T, p.(Gly12Cys) (11%)	pathogenic
4	NM_033360.4(***KRAS***):c.34G>T, p.(Gly12Cys) (42%)	pathogenic
	NM_000222.2(***KIT***):c.55C>T, p.(Arg19Cys) freq 9.6%	VUS
5	NM_000546.5(***TP53***):c.1044G>T, p.(Leu348Phe) (29.1%)	likely pathogenic
6	NM_033360.4(***KRAS***):c.35G>T, p.(Gly12Val) (20.2%)	pathogenic
	NM_000546.5(***TP53***):c.407_424del, p.(Gln136_Cys141del) (20.2%)	likely pathogenic
	NM_203500.1(***KEAP1*):**c.1875A>C, p.(*625Cysext*48) (19.9%)	likely pathogenic
	NM_001270508.2(***TNFAIP3***):c.1034A>T, p.(Tyr345Phe) 10.5%	VUS
7	NM_000417.2(***IL2RA***):c.58C>T, p.(Gln20*) 31%	VUS
	NM_000546.5(***TP53***):c.536A>T (p.His179Leu) (35.9%)	likely- pathogenic
	NM_203500.1(***KEAP1***):c.1408C>T, p.(Arg470Cys) (45.5%)	pathogenic
	NM_000215.3(***JAK3***):c.2112G>T, p.(Leu704Phe)	VUS
	NM_017617.5(***NOTCH1***):c.6168G>A, p.(Met2056Ile) 15.6%	VUS
8	NM_000546.5(***TP53***):c.394A>G (p.Lys132Glu) (18.5%)	likely pathogenic
	NM_017617.5(***NOTCH1***):c.1814A>G, p.(Asn605Ser) 20.8%	VUS
9	NM_033360.4(***KRAS***):c.183A>T, p.(Gln61His) (17.5%)	pathogenic

	NM_000455.5(***STK11***):c.465-1G>A, p.? (21.6%)	likely pathogenic
	NM_001002295.2(***GATA3***):c.820C>G, p.(Leu274Val)	VUS
	NM_001321853.1(***JAK1***):c.1736T>A, p.(Leu579His) 20.6%	VUS
10	NM_017617.5(***NOTCH1***):c.5546del, p.(Asp1849Valfs*38) 6.1%	likley pathogenic
	NM_017617.5(***NOTCH1***):c.1347C>G, p.(Cys449Trp)10.1%	VUS
	NM_000546.5(***TP53***):c.920-1G>A (11.7%)	likely pathogenic
Nonresponders		
11	NM_001321853.1(***JAK1***):c.3290G>A, p.(Gly1097Asp) 9.4%	VUS
	NM_001321786.1(***CBLB***):c.1166A>T, p.(Glu389Val) 6.2%	VUS
	NM_005359.3(***SMAD4***):c.473T>A, p.(Val158Glu) 12.1%	VUS
	NM_001128849.2(***SMARCA4***):c.4168A>C, p.(Lys1390Gln) 10.1%	VUS
12	NM_000546.5(***TP53***):c.1024C>T, p.(Arg342*) (8.4%)	likely pathogenic
	NM_001321853.1(***JAK1***):c.3317A>C, p.(Asn1106Thr) 6.2%	VUS
	NM_001321853.1(***JAK1***):c.3309A>T, p.(Arg1103Ser)6.1%	VUS
	NM_001145661.1(***GATA2***):c.1367C>G, p.(Pro456Arg) 5.6%	VUS
	NM_000222.2(***KIT***):c.2542T>G, p.(Phe848Val) 7.3%	VUS
	NM_005359.3(***SMAD4***):c.473T>A, p.(Val158Glu) 16.1%	VUS
13	NM_000546.5(***TP53***):c.477_497del, p.(Met160_Ser166del) (46.7%)	likely pathogenic
14	ND	
15	NM_000546.5(***TP53***):c.577C>A, p.(His193Asn) (61.2%)	likely pathogenic

cutoff 5% allele frequency, only coding region and splice site +/- 2bp, not including synonymous variants, all variants, heterozygotes
